# Psychometric Properties of the Effort-Reward Imbalance Questionnaire for Teachers (Teacher ERIQ)

**DOI:** 10.3389/fpsyg.2019.02047

**Published:** 2019-09-12

**Authors:** Chuang Ren, Xiying Li, Xuemei Yao, Zhongling Pi, Senqing Qi

**Affiliations:** ^1^MOE Key Laboratory of Modern Teaching Technology, Shaanxi Normal University, Xi’an, China; ^2^Qiqihar Experimental High School, Qiqihar, China; ^3^School of Foreign Languages, Xi’an University of Finance and Economics, Xi’an, China

**Keywords:** effort-reward imbalance, effort-reward imbalance questionnaire for teachers, psychometric properties, teacher, effort-reward imbalance model

## Abstract

The effort-reward imbalance (ERI) model is a theoretical model of a psychosocial work environment with adverse effects on health and well-being that focuses on a mismatch between high efforts spent and low rewards received at work. This study aimed to develop and psychometrically test an effort-reward imbalance questionnaire for teachers (Teacher ERIQ) based on the ERI model. The structure validity, reliability, and criterion validity of the new questionnaire’s scores were evaluated in a sample of 475 Chinese teachers. The results of exploratory factor analysis (EFA) and confirmatory factor analysis (CFA) showed that a structure of four factors of effort (workload, emotional demands, student-related issues, and social responsibility) and two factors of reward (emotional reward and material reward) in accordance with the ERI model had significant factor loadings and acceptable model fit. The Cronbach’s Alpha coefficients of all dimensions’ scores showed that the questionnaire scores had good reliability. Criterion validity was indicated by significant correlation coefficients of scores of most dimensions along with teachers’ self-reported job burnout and non-reciprocal social relations, as well as the ANOVA results showing that the differences of the scores of the two criterion scales in different ERI ratio levels were significant. The results also showed that teacher’s ERI level varied with demographic variables such as age, gender and school type. The Teacher ERIQ is a valid and reliable new measurement for assessing teachers’ psychosocial work characteristics. It can be an important tool to provide new explanations of stress-related health risks among teachers and to guide the development of preventive measurements.

## Introduction

The model of effort-reward imbalance (ERI) first proposed by German medical sociologist Siegrist (1996) has received much attention in occupational health studies because of its predictive power for adverse physical and mental health outcomes (Siegrist and Li, 2016; Rugulies et al., 2017). The model attempts to identify unfavorable psychosocial work characteristics that focus on a mismatch between “costs” and “gains” in costly social transactions. It is based on the notion of social reciprocity at the core of the work contract and asserts that stress occurs if employees feel a mismatch between high cost spent at work and low gain received in turn, and recurrent experience of failed reciprocity elicits sustained negative emotions of reward frustration and related psychobiological stress responses. The model consists of three components: effort, reward and overcommitment. “Effort” means extrinsic work demands. “Reward” is thought to come from three sources: salary or wage (financial reward), career promotion or job security (status-related reward), and esteem or recognition (socioemotional reward). Moreover, a distinct personal pattern of coping with demanding situations termed “overcommitment” is included. Employees characterized by this coping pattern show excessive devotion to work. The theory postulates that overcommitment not only leads to stress response but also amplifies the adverse health effects of ERI (Siegrist, 1996, 2016, 2017).

The effort-reward imbalance model was originally used as a theoretical framework to explain the stress-related risks of company employees who face the threat of uncertain employment and job loss in the context of globalization (Siegrist, 2016). Although most teachers are not facing such risks, they are ideal targets for assessing the utility of ERI because of their occupational particularities. First, the workload of teachers cannot be fully quantified, and the job performance reflected in the students’ academic successes is not immediately visible because it largely depends on the abilities and motivations of students (Kyriacou and Coulthard, 2000). Thus, teachers’ contributions cannot be fully evaluated, recognized, and rewarded by others. Second, this lack of reward is not adequately compensated by the feedback of the students either, because the latter usually depends on factors such as personalities or student preferences, which are not related to the teachers’ contribution (Siegrist, 2017). Finally, the teacher role commonly overemphasizes social responsibility and ideal personality and depreciates individual life value. Sectors of society have high requirements for teachers’ behavioral norms and job performance, while it seems immoral for teachers to mention rewards or even defend basic rights (You and Yang, 2017). Therefore, teachers are often a vulnerable group and in an unequal exchange position in the interaction with students, parents, schools, and even the public. This long-standing non-reciprocal relationship at work has become one of the most important pressures on teachers – it not only has an adverse impact on their psychobiological health but also harms their work performance (Hakanen et al., 2006), which indirectly affects students’ academic, and mental development. Some researchers have examined the applicability of the ERI model in this field (Unterbrink et al., 2007; Lehr et al., 2009, 2010; Zurlo et al., 2010; Loerbroks et al., 2014; Wang et al., 2015; Hinz et al., 2016). Siegrist (2017) conducted a comprehensive review of existing research on the application of the ERI model in education, indicating a high prevalence of ERI at work and elevated risks of poor mental health in teachers, specifically depression and exhaustion.

Although the ERI model has been widely used in this field of different language environments, almost all of the studies used the original employee-based ERI questionnaire or the short version of ERI questionnaire, largely ignoring the differences of work characteristics between employees and teachers. In terms of effort, firstly, teachers face more emotional requirements than physical requirements in their daily work (Unterbrink et al., 2008; Yin and Lee, 2012; Harmsen et al., 2018). They need to pay attention to and understand the emotions of dozens of students, deal with students’ learning and discipline problems, reasonably express their emotions, and use emotions to assist teaching. This may be the reason why mental and psychosomatic diseases are more common in teachers than in non-teachers (Scheuch et al., 2015; Hinz et al., 2016). Secondly, teachers need to conduct student guidance, home visits, homework correction, and other work in their spare time. Their working hours and non-working boundaries are blurred. Lastly, teachers face higher demands from leaders, parents or even the wider public, and bear greater social responsibility in protecting and educating students (You and Yang, 2017). In terms of reward, studies have shown that students’ verbal insults have the strongest impact on teachers’ health relative to other factors (Unterbrink et al., 2008), meaning that students’ cooperation and respect are important to teachers. Therefore, relative to superiors and colleagues, the more important source of socioemotional reward for teachers may be students, parents, and society. In addition, the previous studies showed that middle and high school teachers are threatened by student violence (Guerino et al., 2006; Bauer et al., 2007). It may be suggested that for teachers, job security does not refer to the stability of the work, but to personal safety. For these reasons, the original ERI questionnaire cannot be generalized to teachers. Thus, it is necessary to develop a new questionnaire taking into account the specific efforts given and rewards received for teachers at work.

The theoretical position of overcommitment in the model is constantly evolving with the deepening of the research on ERI theory. The original model did not make a clear distinction between extrinsic and intrinsic effort or examine their respective roles (Siegrist, 1996). In order to highlight the role of individuals’ internal resources, Siegrist (1999) developed the extrinsic effort in the original model into effort and the intrinsic effort into overcommitment. In this view, as an independent concept, overcommitment directly or indirectly affects pressure responses, which means that the component regulates the relationship between ERI and pressure responses. However, the conclusions of relevant research are inconsistent. Some studies have found a moderating effect of overcommitment between ERI and pressure responses such as anger, anxiety, depression, and job satisfaction (Hoggan and Dollard, 2007; Kinman and Jones, 2008; Zurlo et al., 2010), while others did not (Preckel et al., 2007). This study kept overcommitment out of CFA and tested whether overcommitment could regulate the relationship between ERI and health outcomes in teachers.

In addition, previous findings on whether teachers’ ERI is different in demographic variables such as gender and age are inconsistent. Taking German teachers as samples, Unterbrink et al. (2007) found no gender difference but age difference in ERI. Teachers aged 44 and under had lower ERI levels than teachers in the two older age groups. Meanwhile Hinz et al. (2016) study on German teachers showed that female teachers felt a higher level of reward and a lower level of ERI than male teachers, and there was no significant difference in age. One of the most likely reasons for the inconsistencies is that the measurement tools used could not accurately reflect the characteristics of teacher employment. Therefore, this study investigated whether the data collected by the ERI questionnaire reflecting the characteristics of teacher employment would have demographic differences.

This study aimed to develop and psychometrically test a questionnaire that reflects teachers’ unique psychosocial work environment, focusing on a balance between effort and reward in teaching. Psychometric properties were mainly tested from several aspects: First, the structure validity was tested by exploratory factor analysis (EFA), confirmatory factor analysis (CFA), and calculating item-total correlation and inter-item correlation. Second, the reliability was tested by calculating Cronbach’s Alpha coefficients and coefficients of stability. Third, the criterion validity was tested by calculating the correlation between scores of ERI and teachers’ subjective report of job burnout and non-reciprocal relationship. In addition, the role of overcommitment on the relationship between ERI and job burnout as well as the difference of ERI in demographic variables were also investigated.

## Materials and Methods

### Phase I: Development of the Effort-Reward Imbalance Questionnaire for Teachers (Teacher ERIQ)

The items on Teacher ERIQ were designed in the Chinese language in a way to preserve the original meaning while capturing the specific characteristics of an adverse psychosocial environment for teachers. In compliance with the authors of the original ERI scale, each item was evaluated for its ability to reflect the distinct work environment of the teacher group. The items of overcommitment do not involve occupational particularity and are also applicable to teachers, so the overcommitment scale in the original ERI questionnaire is still used, and the effort and reward scale is developed here. Initially, we conducted a review to identify the related aspects of effort and reward in the field, as mentioned in the introduction; the results showed that efforts mainly involve four aspects: workload (“heavy workload,” “work overtime,” and “blurred working and non-working time boundaries”), social responsibility, emotional requirements, and student-related issues (“students” learning and behavioral issues”). Rewards mainly involve two aspects: mental rewards (respect and recognition from school, students, parents, and society) and material rewards (money, career promotion, and job security). Then we designed an interview outline based on the theoretical structure of ERI and the results of literature review and interviewed four teachers recruited from primary and secondary schools. The results verified the theoretical hypothesis of the subdimensions of effort and reward proposed above, further clarified and supplemented the contents of each subdimension and provided specific expressions as follows: “Something that takes up time but not very helpful for teaching, such as too frequent and formalistic assessments” was added in the workload; “Feeling constrained in disciplining students” and “lack of family education” were added in the student-related issues; and emotional requirements are reflected in “constant worry,” “emotional exhaustion,” and “bad mood.” In addition to respect and recognition from others, self-worth is also an important source of emotional reward.

By comparing the results with the original ERI questionnaire, it can be seen that 7 of the 17 items of the original questionnaire still applied to teachers and were retained (e.g., “I have constant time pressure due to a heavy workload”). Four items were deleted (e.g., “I have many interruptions and disturbances in my job”) and 6 items were modified (e.g., “physically demanding” replaced with “emotionally demanding”) because they did not match with this context to varying degrees. There are also 15 new items reflecting the particularity of the profession that need to be added (e.g., “I am often pressured by students’ learning problems”). Based on the above results, the items were generated after the argumentation of experts in the field of development and educational psychology, mainly based on two criteria: one is to evaluate whether each item accurately reflects the connotation of its dimension, the other is to check whether the expression of the item is accurate, clear, and easy to understand for teachers. The items were then evaluated by several recruited teachers and slightly modified in expression. A preliminary version of Teacher ERIQ was developed with 32 items in total. Effort is measured by 17 items, divided into four subdimensions: workload (5 items), social responsibility (4 items), emotional demands (4 items), and student-related issues (4 items). Reward is measured by 15 items, divided into two subdimensions: emotional reward (8 items), and material reward (7 items).

In the two rounds of testing, the items were examined by item analysis and EFA. In the item analysis, the following criteria are considered for the retention, modification or elimination for the items: (1) item-total correlation is significant; (2) discrimination ratio is significant; (3) commonality is higher than 0.16; and (4) factor loading is higher than 0.4. In the EFA, the following criteria are considered comprehensively: (1) the load of each factor exceeds 0.4; (2) each item does not have multiple loads – that is, it cannot load more than 0.4 on two or more factors at the same time – and if the load of the item exceeds 0.4 on both factors, but the load difference is greater than 0.15, the item is also retained; and (3) the number of items included in every factor is greater than or equal to 3.

The first round of testing was conducted with 155 teachers recruited from a teacher training course in Xi’an, a city of China, including local elementary, middle, and high school teachers. The item analysis results are acceptable (range item-total correlations = 0.324–0.628, *p* < 0.001; range discrimination ratio = 2.718–9.211, *p* < 0.001; range commonality = 0.160–0.523; range factor loading = 0.400–0.702). EFA was made on the effort and reward, respectively. The KMO values of the two parts are 0.886 and 0.809 and the spherical tests are significant, indicating that both are suitable for factor analysis. After principal component analysis and oblique rotation, factors with eigenvalues greater than 1 are extracted. The results showed that the effort was divided into four factors, which accounted for 58.05% of the variation. Factor loadings range from 0.387 to 0.832. The factor composition is generally consistent with the hypothesis, but there are five items entering factors that are different from the hypothesis, so the expression of them was modified. For example, e36 (“At work, I often need to spend a lot of energy to adjust my mood”) was loaded on both the workload dimension (0.582) and the student-related issues dimension (0.444), and it was intended to be classified as an emotional demand dimension. Its expression emphasizes “spending a lot of energy” and may be the reason for entering the workload dimension, so it was modified as “At work, I often need to adjust my mood.” In addition, E21 (“In my work, I often have to do something not very helpful for teaching”) was loaded on both the student-related issues dimension (0.387) and the emotional demands dimension (0.320), but the load on each factor was less than 0.4, so it was deleted. The reward was divided into two factors, accounting for 41.61% of the variance. Factor loadings ranged from 0.412 to 0.774, and the factor composition was consistent with the hypothesis. Thus, the modified questionnaire included 31 items. The revised items were evaluated by several recruited teachers and slightly modified to conform to the real situation and expression habits of the teachers.

In the second round of testing, 442 teachers recruited from primary, middle and high schools in Xi’an participated in the test of the modified questionnaire. The item analysis results were acceptable (range item-total correlations = 0.366–0.575, *p* < 0.001; range discrimination ratio = 7.291–14.364, *p* < 0.001; range commonality = 0.191–0.518; range factor loading = 0.437–0.719) except for e26 (“The public lacks understanding of teachers”), which had a commonality of 0.115 and a factor loading of 0.340, so the item was deleted. EFA was made on the effort and reward. The KMO values of the two parts were 0.882 and 0.837, and the spherical tests were significant, indicating that both were suitable for factor analysis. After the same procedures as the first round, the results showed that the effort was divided into four factors, which accounted for 53.71% of variation. Factor loadings ranged from 0.358 to 0.793. The factor composition was consistent with the hypothesis except that two items had unknown dimensions. E31 (“I am often pressured to students’ behavior problem”) had double loads and it was intended to be classified as the student-related issues dimension (0.358) but was classified into the workload dimension (0.470). E35 (“At work, I often need to adjust my mood”) had double loads and was intended to be classified as emotional demands (0.445) but was classified into the workload dimension (0.506). Since there is no obvious ambiguity in the expression of the two items and considering the balance of the number of items among the dimensions, the two items were removed. The reward was divided into two factors, accounting for 40.43% of the variance. Factor loadings ranged from 0.496 to 0.749, and the factor composition was consistent with the hypothesis. The revised items were evaluated by several recruited teachers and slightly modified to conform to the real situation and expression habits of the teachers, thus forming the final questionnaire.

So the final version of Teacher ERIQ was developed with 28 items in total. Effort is measured by 14 items, divided into four subdimensions: workload (4 items), social responsibility (4 items), emotional demands (3 items), and student-related issues (3 items). Reward is measured by 14 items, divided into two subdimensions: emotional reward (7 items) and material reward (7 items). The workload contains time pressure, work overtime and blurred working and non-working time boundaries. Social responsibility refers to the responsibilities and requirements from significant parties. Emotional demands refer to the exhaustion, worries and negative emotions caused by work. The student-related issues contain students’ learning and behavioral issues, feeling constrained in disciplining students, and lack of family education. Emotional reward includes respect from students, parents, schools and society, as well as teachers’ self-worth. Material reward includes salary, the promotion of professional title, and job security.

### Phase II: Study Design and Participants

#### Design

The cross-sectional survey was designed and conducted to examine the psychometric properties of the effort-reward imbalance questionnaire for teachers (Teacher ERIQ). Four hundred and seventy-five teachers were recruited from primary, middle, and high schools in two cities in China, Xi’an and Xinxiang, in 2018. The study protocol was approved by the Ethical Committee of the Shaanxi Normal University.

Participants completed or submitted the following: (1) effort-reward imbalance questionnaire for teachers (Teacher ERIQ); (2) Overcommitment questionnaire; (3) Maslach burnout Inventory-Educator’s Survey (MBI-ES); (4) Non-Reciprocal Social Relations Questionnaire; and (5) socio-demographic information including sex (male/female), age (in years), and type of school (primary school/junior high school/high school).

#### Participants

Four hundred and forty-two teachers completed questionnaires (response rate ≥90%). Three hundred and seventeen of them were females (71.4%), one hundred and twenty-two were males (27.5%), and five were missing. The age ranged between 24 and 51 years (mean age 37.19). They were representative of the different types of school according to the age of pupils: 196 primary school teachers (44.1%), 125 junior high school teachers (28.1%), 99 high school teachers (22.4%), and 24 were missing.

#### Measurements

##### Effort-reward imbalance questionnaire for teachers (Teacher ERIQ)

For measuring ERI in teachers the newly developed questionnaire was used. Effort contains 14 items including four subdimensions (workload, student-related issues, emotional demands, and social responsibility), and reward contains 14 items including two subdimensions (emotional reward and material reward). Each item can be scored with values between 1 and 5 and a sum score of these ratings was constructed as the unidimensionality of the scale. Thus, the total sum score based on the 14 items measuring effort and reward varies between 14 and 70. The higher the score of effort, the higher the teacher’s perceived demands. The lower the score of reward, the lower the teacher’s perceived reward.

In line with Siegrist et al. (2004), the effort-reward ratio was computed for every respondent according to the formula *e/*(*r* × *c*) where “*e*” is the sum score of the effort scale, “*r*” is the sum score of the reward scale and “*c*” defines a correction factor for different numbers of items in the nominator and denominator, so the correlation factor of the newly developed questionnaire is 1 (14/14). Therefore, a value close to zero indicates a favorable condition (relatively low effort, relatively high reward), whereas a value beyond 1.0 indicates an ERI, e.g., a high amount of effort spent that is not met by the rewards received or expected in turn. As a predictor of burnout, this ratio was transformed into a binary variable (values ≤1 vs. >1). In order to differentiate teachers with slight, moderate and severe imbalance, teachers with a ratio >1 were divided into three groups, namely: (1) ratio score ≤1.5 (slight imbalance), (2) ratio score ranging from 1.5 to 2 (moderate imbalance), and (3) ratio score reached values above 2 (severe imbalance).

##### Overcommitment

The overcommitment scale in the original ERI questionnaire was used. It contains 6 items that refer to the intrinsic involvement of employees in their work, reflecting the excessive participation in work, and the inability to pull away from work responsibilities. Each item can be scored with values between 1 and 5, and a total sum score based on the 6 items varies between 6 and 30. The higher the score, the more difficult it is for teachers to get away from work. The Cronbach’s alpha coefficient in this study is 0.738.

##### Maslach burnout inventory-educator’s survey (MBI-ES)

This study used a questionnaire based on the localization of the MBI-Educator’s Survey (MBI-ES; Maslach et al., 1986) by Xin-chun et al. (2016). A total of 22 items are divided into three dimensions: emotional exhaustion (8 items), depersonalization (6 items), and low personal achievement (8 items). Participants responded to items using 7-point Likert scale ratings from “never” to “always.” The higher the total score, the more serious the burnout. In this study, the Cronbach’s Alpha coefficient of the scale score is 0.807, and the Cronbach’s Alpha coefficient of the subdimensions’ scores is 0.627–0.884.

##### Non-reciprocal social relations questionnaire

The items in the Non-reciprocal Social Relations Questionnaire (von demKnesebeck and Siegrist, 2003) that did not meet the teachers’ situation were deleted, and the object in the items was changed from “partner or child” to “student.” The revised scale has 9 items. Participants responded to items using a 5-point Likert scale rating from “strongly disagree” to “strongly agree” (von demKnesebeck and Siegrist, 2003; Chandola et al., 2007). The higher the total score, the more unequal the exchange between teachers and students. The Cronbach’s alpha coefficient in this study is 0.615. CFA results showed that the measurement model fits well (χ^2^/df = 1.794, TLI = 0.870, CFI = 0.906, SRMR = 0.057, RMSEA = 0.074).

#### Analysis

Firstly, in order to examine the psychometric properties of Teacher ERIQ, we tested (1) structure validity – the factorial structure of the questionnaire was tested by EFA, CFA, and calculating factor-total Pearson’s *r* correlations and Pearson’s *r* correlations between factors. A moderate or high factor-total correlation indicates that the factor reflects the total well. A low or moderate correlation between factors indicates that they are independent and there are no higher order ones. In the development of the questionnaire, samples collected are usually divided into two parts, half of which is used to explore the model structure (EFA) while the other half is used to verify whether the conclusion of the exploration is correct or not (CFA). Therefore, in this study, half of the samples were randomly selected for conducting EFA by IBM SPSS 22 and the other half for CFA. The criteria of the former are the same as those in phase I. There are two main criteria for CFA, one is that the factor loadings are higher than 0.4, and the other is to meet the following model fitting indexes: χ^2^/*df* < 3, Tucker-Lewis Index (TLI > 0.9), comparative fit index (CFI > 0.9), Akaike information criterion (AIC), Bayesian information criterion (BIC), standardized root mean square residual (SRMR < 0.08), and root mean square error of approximation (RMSEA < 0.08). AIC and BIC are used for comparative comparison. The smaller the two indexes are, the more frugal the model is. For a good test EFA and CFA results often have consistency and stability. We then tested (2) reliability – the internal consistency of the questionnaire scores was tested by calculating Cronbach’s alpha coefficients. In addition, 90 teachers were recruited and answered the questionnaire twice in 2 weeks. The test-retest reliability was tested by calculating the correlation between the scores of the questionnaire twice. (3) The criterion validity was investigated by computing Pearson’s *r* correlation coefficients between scores of ERI and teachers’ self-reported job burnout and *r* between scores of ERI and non-reciprocal social relations. Further, analyses of covariance (ANOVA) were carried out with job burnout and non-reciprocal social relations as the dependent continuous variables and ERI scores as independent variable at four levels.

Secondly, we tested the moderating effect of overcommitment on relationship between ERI and burnout by hierarchical linear regression analysis. Thirdly, we tested the distribution of ERI scores (ERI ratio; effort, reward and their subdimensions; overcommitment) by calculating statistical parameters and the frequency of the ERI scores at varying levels. Finally, we examined whether the ERI scores differ with respect to socio-demographic factors, i.e., age, gender, and type of school, with ERI scores as the dependent and sociodemographic factors as the independent variables.

CFA was performed by MPLUS 7, and other statistical analyses were performed by IBM SPSS 22.

## Results

### Psychometric Properties of Teacher ERIQ

Based on the ERI framework, the Teacher ERIQ was developed and finalized in phase I and psychometrically tested in phase II of the study.

(1) Structure validity: The structure validity of scores of the questionnaire with 28 items was tested by EFA and CFA as well as calculating factor-total Pearson’s *r* correlations and Pearson’s *r* correlations between factors. The results of factor-total Pearson’s *r* correlations showed that subdimensions of effort were moderately or highly correlated with effort and ERI ratio (0.491–0.815), and subdimensions of reward were moderately or highly correlated with reward and ERI ratio (0.755–0.885); both indicated that factors measure effort, reward, and ERI well. The results of Pearson’s r correlations between factors showed that there were low or moderate correlations (0.274–0.530) between subdimensions of effort, and there was moderate correlation (0.510) between subdimensions of reward; both indicated that factors were related and not the same factor. See Supplementary Tables 1, 2 for detailed results.

Table 1 shows the results of EFA which was made on the effort and reward, respectively. The KMO values of the two parts were 0.858 and 0.835, and the spherical tests were significant, indicating that both were suitable for factor analysis. After the same procedures as in phase I, the results showed that the effort was divided into four factors, which accounted for 65.10% of variation. Factor loadings ranged from 0.476 to 0.828. The reward was divided into two factors, which accounted for 41.40% of variation. Factor loadings ranged from 0.312 to 0.774.

**TABLE 1:** The standardized factor loadings, Cronbach’s Alpha for factors and items (*N* = 216).

**Factors & items**	**Standardized factor loading**	**Eigen value**	**% of variance**	**Cronbach’s alpha**	**Coefficient of stability**
Effort			65.101	0.844	0.741^∗∗^
Workload		5.230	37.355	0.812	0.683^∗∗^
e36	0.828				
e27	0.795				
e23	0.781				
e32	0.563				
Social responsibility		1.583	11.309	0.765	0.720^∗∗^
e2	0.809				
e17	0.799				
e19	0.745				
e8	0.578				
Emotional demands		1.424	10.170	0.708	0.675^∗∗^
e1	0.803				
e10	0.694				
e37	0.652				
Student-related issues		0.877	6.267	0.594	0.608^∗∗^
e5	0.803				
e6	0.636				
e21	0.476				
Reward			41.397	0.819	0.678^∗∗^
Material reward		4.333	30.951	0.714	0.648^∗∗^
e3	0.706				
e30	0.644				
e22	0.610				
e18	0.582				
e16	0.560				
e4	0.512				
e7	0.501				
Emotional reward		1.462	10.446	0.785	0.678^∗∗^
e29	0.312				
e24	0.774				
e28	0.711				
e13	0.710				
e11	0.564				
e14	0.525				
e34	0.522				
ERI					0.699^∗∗^

*^∗∗^*p* < 0.01.*

In effort, in addition to the four-factor model (E4, including workload, social responsibility, student-related issues, and emotional demands) assumed in this study, the single-factor model (E1) used in previous studies was also tested (Siegrist et al., 2004; Sperlich et al., 2012). Considering that the eigenvalue of the student-related issues is less than 1, we deleted this dimension and got a three-factor model (E3, including workload, social responsibility, and emotional demands). In reward, in addition to the two-factor model (R2, including emotional reward, and material reward) assumed in this study, the single-factor model (R1) used in previous studies was also tested (Siegrist et al., 2004).

Table 2 shows the goodness-of-fit indexes of the five models. The results of the normality test showed that some items had substantial non-normal distributions in all five models, so we adopted maximum likelihood method (MLM). It can be seen that the four models of E3, E4, R2, and R1 were qualified. Considering all indicators, R2 is better than R1, and E4 is close to E3. However, from the reduction degree of the important results of literature review and interviews, E4 is obviously better than E3. So, the four-factor model was the best-fit model for effort and the two-factor model was the best-fit model for reward, which validated the hypothesis of this study.

**TABLE 2:** Goodness-of-fit indexes of the five hypothetical models tested in the CFA.

**Model**	**χ^2^**	**df**	**χ^2^/df**	**TLI**	**CFI**	**AIC**	**BIC**	**SRMR**	**RMSEA (90% CI)**
E4	128.847	71	1.81	0.914	0.933	7136.822	7300.795	0.063	0.060 (0.043 0.077)
E3	87.099	41	2.12	0.915	0.936	5191.695	5314.994	0.065	0.070 (0.050 0.091)
E1	312.702	77	4.06	0.677	0.727	7336.097	7479.573	0.088	0.117 (0.103 0.130)
R2	146.436	76	1.93	0.873	0.894	7918.862	8063.599	0.059	0.066 (0.050 0.082)
R1	179.205	77	2.33	0.818	0.846	7953.178	8094.549	0.066	0.082 (0.067 0.097)

**FIGURE 1:**
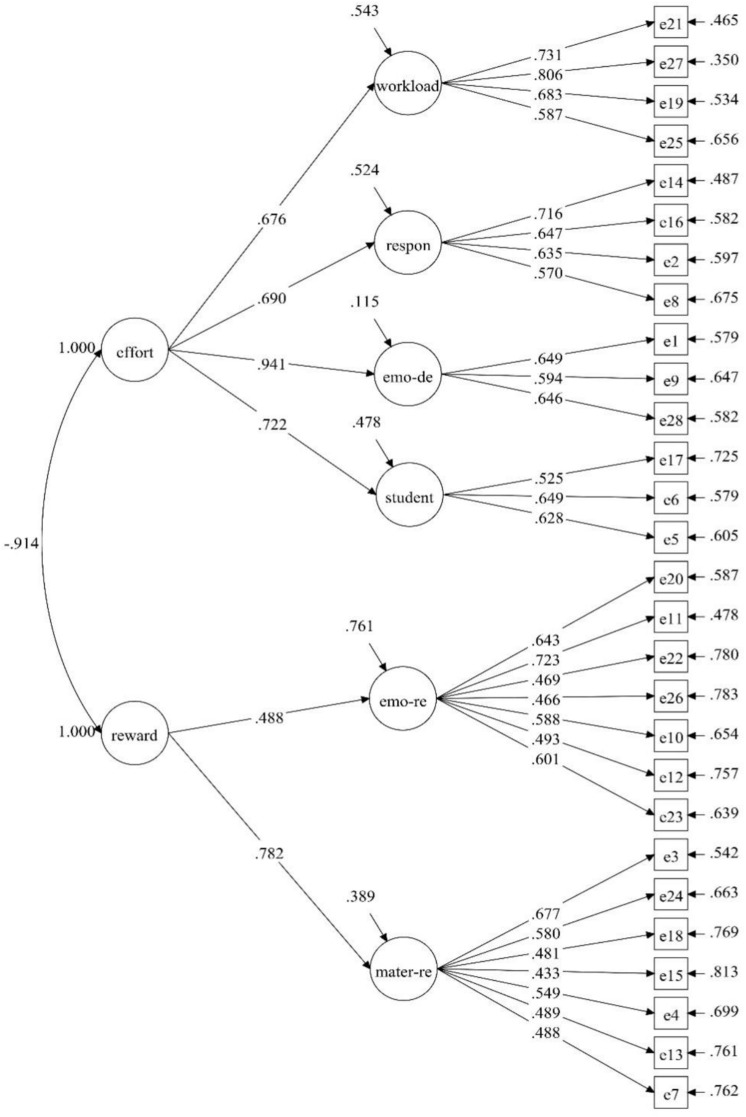
Factorial structures of Teacher ERIQ.

Figure 1 shows the model of four factors of effort (workload, emotional demands, student-related issues, and social responsibility) and two factors of reward (emotional reward and material reward) aligning with the theoretical structure of the ERI framework. For the same reason as above, the MLM parameter estimation method was also adopted. The goodness-of-fit indexes were excellent and all factors had high standardized factor loadings (0.433–0.806).

(2) Reliability: as shown in Table 1, Cronbach’s Alphas of ERI scores were satisfactory, ranging from 0.590 to 0.840. Considering that the errors of the factors are not correlated, the internal consistency and reliability are not lower than this range (Wen and Bao-Juan, 2011). The test-retest reliability is acceptable with coefficients of stability ranging from 0.608 to 0.741.

(3) Criterion validity: Table 3 shows the Pearson’s *r* correlations among ERI, job burnout, non-reciprocal social relations and the subdimensions of the first two. As expected, all of the ERI scores showed significant moderate or high correlations with job burnout (0.273–0.591), and most of the ERI scores showed significant moderate correlation with non-reciprocal social relations.

**TABLE 3:** Pearson’s *r* correlation coefficients among ERI scores, job burnout scores, and non-reciprocal social relations.

	**Emotional exhaustion**	**Reduced personal accomplishment**	**Depersonalization**	**Job burnout**	**Non-reciprocal social relations**
					
Emotional demands	0.633^∗∗^	−0.031	0.137	0.470^∗∗^	0.221^∗^
Student-related issues	0.487^∗∗^	0.195^∗^	0.116	0.471^∗∗^	0.254^∗∗^
Workload	0.640^∗∗^	0.076	0.284^∗∗^	0.591^∗∗^	0.170
Social responsibility	0.430^∗∗^	−0.114	0.119	0.273^∗∗^	0.141
Emotional reward	−0.267^∗∗^	−0.168	−0.172	−0.328^∗∗^	−0.393^∗∗^
Material reward	−0.555^∗∗^	−0.054	−0.116	−0.484^∗∗^	−0.331^∗∗^
Effort	0.702^∗∗^	0.014	0.212^∗^	0.576^∗∗^	0.210^∗^
Reward	−0.480^∗∗^	−0.133	−0.150	−0.468^∗∗^	−0.438^∗∗^
ERI	0.564^∗∗^	0.026	0.152	0.480^∗∗^	0.377^∗∗^

*^∗^*p* < 0.05; ^∗∗^*p* < 0.01.*

Table 4 shows that teachers’ job burnout and non-reciprocal relationship were significantly different in different degrees of ERI ratio.

**TABLE 4:** ANCOVA results.

	**ERI ratio**	***N***	***M***	***SD***	***F***	***Post hoc***
Job burnout	1(≤1)	12	55.417	7.914	18.826^∗∗∗^	1 < 2 < 3, 4
	2(1.01-1.5)	51	73.686	12.056		
	3(1.51-2)	28	80.321	8.429		
	4(≥2.01)	7	88.143	14.147		
Non-reciprocal social relations	1(≤1)	13	25.077	3.095	6.169^∗∗^	1, 2 < 3, 4
	2(1.01-1.5)	55	25.400	3.071		
	3(1.51-2)	30	27.833	4.202		
	4(≥2.01)	8	29.625	3.583		

*1, no ERI; 2, slight ERI; 3, moderate ERI; 4, severe ERI. The goodness-of-fit indexes: TLI = 0.801, CFI = 0.818, SRMR = 0.081, RMSEA (90% CI) = 0.059 (0.052 0.067), AIC = 15445.166, BIC = 15748.803, and χ^2^/df = 1.787 (*N* = 226). Respon, social responsibility; emo-de, emotional demands; student, student-related issues; emo-re, emotional reward; mater-re, material reward. ^∗∗^*p* < 0.01; ^∗∗∗^*p* < 0.001.*

### The Role of Overcommitment on the Relationship Between ERI and Job Burnout

After centralization, ERI and overcommitment were multiplied to obtain the interaction. After adjustment for gender, age and types of school, the results showed that the main effect and interaction effect of ERI and overcommitment were significant (see Table 5). A high level of burnout was associated with high ERI and high overcommitment, while a high level of overcommitment reduced the positive effect of ERI on job burnout.

**TABLE 5:** Results of the hierarchical linear regression analysis.

**Variables**	**Burnout**		
	**Step 1 (β)**	**Step 2 (β)**	**Step 3 (β)**
Sex	−3.015	−1.002	–0.310
Dummy 1	−5.102^∗∗^	−2.204	–2.295
Dummy 2	−2.082	−0.520	0.024
Age	−0.124	−0.203^∗^	–0.170
ERI		15.483^∗∗∗^	17.698^∗∗∗^
Overcommitment		0.568^∗∗^	0.385^∗^
ERI^∗^overcommitment			–0.977^∗∗^
*R*^2^	0.037	0.391	0.403
ΔR^2^	0.037^∗∗^	0.353^∗∗∗^	0.013^∗∗^

*Dummy 1, “primary school” vs. “junior high school”; Dummy 2, “high school” vs. “junior high school.” ^∗^*p* < 0.05; ^∗∗^*p* < 0.01; ^∗∗∗^*p* < 0.001.*

### Prevalence of ERI Among Teachers

The statistical parameters showed that the mean value for the ERI ratio was 1.519, indicating a relatively common imbalance between teachers’ effort and reward in work. See Supplementary Table 3 for detailed results. The frequency distribution of ERI scores showed that 92.1% of teachers perceived lack of reciprocity, that is, they pay more than they receive in turn. Most (77%) teachers pay 1 to 2 times the rewards they receive. It was mainly caused by high effort. A total of 61.7% of teachers’ effort was at a high level (categories 4 and 5 of response format) and the most serious subdimension was social responsibility, with a high level of 92.6%. A total of 7.4% of teachers’ reward was at a low level (categories 1 and 2 of response format), and low material reward was the main reason, with a low level of 39.2%. With respect to overcommitment, 57.5% of teachers reached a high level, suggesting an unfavorable psychosocial condition. See Supplementary Table 4 for detailed results.

### Associations Between ERI Components and Sociodemographic Characteristics

The results of sociodemographic differences in ERI scores were as follows. In terms of gender difference, male teachers’ scores of workload, social responsibility and effort were significantly higher than female teachers, their scores of material reward and reward were significantly lower than female teachers, and their ERI level was significantly higher than female teachers. In terms of age difference, teachers aged 30 and under had significantly lower workload scores than the other four groups. In terms of school type difference, the workload of high school teachers was significantly higher than that of middle school and primary school teachers; junior high school teachers had significantly higher student-related issues than primary and high school teachers; primary school teachers’ emotional reward and reward scores were significantly higher than junior high school teachers; and the ERI degree of junior middle school teachers was significantly higher than that of primary school teachers. In a word, ERI scores showed significant differences in gender, age and school type. See Supplementary Table 5 for detailed results.

## Discussion

This study aimed to develop and psychometrically test an Teacher ERIQ based on the ERI model. The results indicated that the Teacher ERIQ scores had good overall validity and reliability for use among teachers. It also proved the moderating effect of overcommitment on the relationship between ERI and job burnout and showed that teachers’ ERI level varied with age, gender and school type. To our knowledge, this is the first study based on the ERI model to develop a questionnaire reflecting the unique psychosocial work environment of teachers.

In the development stage of the questionnaire, each item was evaluated for its ability to reflect teachers’ distinct psychosocial work environment, focusing on effort and reward, and the content validity was guaranteed. This was mainly reflected in two processes: In the early stage of item generation, the methods of relevant literature review, teacher interview, and expert argument were adopted to ensure that the content reflected in the questionnaire was accurate and comprehensive. In addition, after each revision, the items were evaluated by teachers and modified until they could reflect the real situation of teachers and conform to teachers’ expression habits. At the same time, the structure validity of the questionnaire scores was improved in the two rounds of testing. The expression of several items was adjusted and four items were deleted according to the results of item analysis and EFA. Afterward, which factor each item belonged to was more explicit. Factor loadings and variances explained of items were both improved. Thus, revisions to the initial 32-item version of the Teacher ERIQ substantially improved the structure validity.

In the test stage of the questionnaire, five hypothetical models were tested by CFA, and the four-factor model (workload, emotional demands, student-related issues, and social responsibility) was the best-fit model for effort, and the two-factor model (emotional reward and material reward) was the best-fit model for reward, which validated the hypothesis of this study. Further, the results of EFA and CFA validated the structure of four factors of effort and two factors of reward, aligning with the theoretical structure of the ERI framework. This is different from the previous studies that revised ERI scale, most of which believed that effort was a single factor (Fukuda et al., 2010; Sperlich et al., 2012). The main reason might be that teachers’ work had prominent occupational particularities such as emotional requirements and students’ problems (Montgomery and Rupp, 2005; Guerino et al., 2006; Bauer et al., 2007; Mahan et al., 2010). In particular, a study revising the ERI for reservists on a military deployment put overcommitment into the model and divided the effort into internal (overcommitment) and external (time and energy) effort (Lang et al., 2010). Soldiers are obligated and disciplined, and even if they do not care about work, their external effort may not be reduced. Their external and internal effort do not affect each other and are relatively independent. However, there may be a significant difference in the level of external effort between teachers with high overcommitment and those with low overcommitment. Therefore, considering the difference of the nature of occupation, this study did not include overcommitment into the effort. Previous studies mostly classified reward in the following ways: (1) Classify by nature, such as economic, status, and emotional reward (Siegrist et al., 2004); (2) classify by source, such as internal and external (Lang et al., 2010); and (3) classify by the object of the source, such as oneself, society, partners and children (Sperlich et al., 2012). However, for the convenience and timeliness of the questionnaire, the number of items was limited. Therefore, combined with the results of EFA, the reward in this questionnaire was divided into emotional reward and material reward.

The Cronbach’s Alpha coefficients and coefficients of stability of all dimensions’ scores were satisfactory. The results of correlation and ANOVA verified the criterion validity of the questionnaire scores. Further, we found that the correlation between ERI and job burnout was mainly caused by the correlation between ERI and emotional exhaustion, while little was explained by deindividuation, and low personal achievement. This suggests that the imbalance between pay and return in teachers’ work may directly cause their emotional exhaustion, but it will not directly affect their self-evaluation and basic attitude toward students and work. In addition, among all dimensions of the questionnaire, only student-related issues were significantly related to reduced personal achievement. Thus, it can be speculated that problems related to students may lead to teachers’ negative self-evaluation, thus reducing their sense of personal accomplishment. This is consistent with the results of Einar M. Skaalvik and Skaalvik (2007) research on teachers’ self-efficacy, which show that the structure of teachers’ self-efficacy is largely related to students.

In this study, overcommitment was considered as an independent individual trait that may have a direct or indirect impact on stress responses. The results of hierarchical linear regression analysis proved the moderating effect of overcommitment on the relationship between ERI and job burnout, which was consistent with previous studies (Kinman and Jones, 2008; Zurlo et al., 2010). However, it is worth noting that the delta R-square is less than 3%, which may be influenced by other important factors in the process of the imbalance of teachers’ effort and reward into the stress response. So, the interpretation of the result needs to be cautious.

Descriptive statistics indicated that the imbalance between teachers’ effort and reward is relatively common, which is mainly because of high effort. It showed that teaching is a highly demanding profession, which is consistent with the previous research results on teachers’ work pressure (Guerino et al., 2006; Bauer et al., 2007; Mahan et al., 2010). The results also showed a lower level of material reward relative to emotional reward, suggesting possible defects in the relevant systems and policies of teacher treatment.

Our analyses have shown that ERI differs according to socio-demographic factors. There was gender difference in ERI of the teacher. Male teachers experience higher effort, lower reward and higher ERI than female teachers, which is consistent with the research results of Hinz et al. (2016). This may be due to the different social division of labor. Specifically, women’s identification of their own value comes from work and family; however, men’s generally comes from work, and they may equate the income and social status brought by work with their own value, so they have high expectations for work rewards (Zhao et al., 2019). When the actual situation cannot meet the expectation, the sense of giving is high and the sense of reward is low, which easily leads to the ERI.

There was age difference in workload of the teacher. Teachers aged 30 and below had significantly lower workload scores than the other four groups, which is consistent with the research results of Unterbrink et al. (2007). Skaalvik and Skaalvik (2015) reported that although teachers at different ages experienced the same stressors at school, older teachers needed more time to recover from stress.

There were differences in ERI scores of teachers among different types of schools. Firstly, the workload of high school teachers is significantly higher than that of other groups. Secondly, junior high school teachers have significantly higher student-related issues than other groups. Thirdly, primary school teachers have significantly higher emotional reward than junior high school teachers. There are two possible main reasons for the heavy workload of high school teachers. One is that the curriculum of senior high school is obviously more demanding than that of the two other types of school in both depth and breadth, and the other is that they are under heavy pressure from college entrance examination (Skaalvik and Skaalvik, 2017). The reason for the high level of student-related issues in junior high school teachers may be that the students they teach are in adolescence. Junior high school students are more emotional and self-centered compared with primary school students and lack appropriate coping skills to deal with daily stressors compared with high school students (McMahon et al., 2013). Therefore, junior high school teachers are faced with more problems relating to students’ emotion, behavior and habit formation and have a higher possibility of conflict with students as well as heavier emotional exhaustion. The reason for the high level of emotional reward of primary school teachers may be that primary school students are naiver, have fewer emotional problems, and express emotions more directly compared with junior high school students.

The value of this study is mainly reflected in the following points. Firstly, as far as we know, this is the first questionnaire developed based on the ERI model to reflect teachers’ social psychological work characteristics. The questionnaire provides an effective measurement tool for the ERI model’s application in the field of teachers, so the research results can more accurately reveal the teachers’ ERI, thus providing an empirical basis for interventions to improve teachers’ employment conditions. Secondly, in order to build a questionnaire structure reflecting teachers’ psychosocial work characteristics while conforming to the original ERI model on the whole, we revised the questionnaire for several times during the development of the questionnaire, and EFA and CFA are combined to ensure the structural validity and content validity of the questionnaire scores in the process of testing the psychological characteristics of the questionnaire.

Some limitations of the study need to be addressed. Firstly, the findings come from a cross-sectional design study, so the ERI scores’ criterion validity, especially its predictive power of job burnout, needs to be further confirmed by more longitudinal design studies. Secondly, the criterion used in this study is subjective questionnaires, and individual differences in personality traits may affect the report of job burnout and non-reciprocal social relations. Future studies should be combined with more objective criteria such as data observed and evaluated by others or clinical diagnosis. Thirdly, this study only investigated teachers in two provinces of China, and the generalization of the results needs to be analyzed on a case-by-case basis. Finally, although the regulatory effect of overcommitment in this study is significant, its effect size is low. The specific factors that may exist in this mechanism need to be further studied.

## Conclusion

In conclusion, it can be stated that the newly developed Teacher ERIQ is a reliable and valid measurement instrument that can be used to measure teachers’ psychosocial traits focusing on a mismatch between effort spent and reward received in costly social transactions. This questionnaire could serve as a tool to discover the important psychosocial work characteristics and explain stress-related health risks among teachers so as to provide the starting point for investment in teachers’ work health in relevant departments and schools. In addition, it can also provide a self-examination tool for teachers to know their views on efforts and rewards at work so as to inspire them to change their unhealthy cognition, acquire a sense of value and reward from work in various aspects and improve adverse mental health condition.

## Data Availability

The datasets generated for this study are available on request to the corresponding author.

## Ethics Statement

The studies involving human participants were reviewed and approved by the Shaanxi Normal University. The patients/participants provided their written informed consent to participate in this study.

## Author Contributions

CR and XL contributed to the design and planning of the experiment, data analyses, and writing of the manuscript. ZP took part in the planning and designing of the experiment, data analyses, and manuscript preparation. XY and SQ contributed to the design and planning of the experiment. All authors read and approved the final manuscript.

## Conflict of Interest Statement

The authors declare that the research was conducted in the absence of any commercial or financial relationships that could be construed as a potential conflict of interest.
