# Supplementary material for: Psychometric Properties of the Effort-Reward Imbalance Questionnaire for Teachers (Teacher ERIQ)
Source: Front Psychol. 2019 Sep 12;10:2047. doi: 10.3389/fpsyg.2019.02047 (PMC6751258; doi:10.3389/fpsyg.2019.02047)
Supplement: Supplementary file 1 [file Data_Sheet_1.zip › tables/TABLE 1 Pearson’s r correlations between the subdimensions of effort.doc]

**TABLE 1** Pearson’s r correlations between the subdimensions of effort

|  | Emotional  demands | Student-related issues | Workload | Social responsibility | ERI |
| --- | --- | --- | --- | --- | --- |
| Effort | 0.815^**^ | 0.723^**^ | 0.765^**^ | 0.715^**^ | 0.731^**^ |
| Emotional demands |  | 0.490^**^ | 0.530^**^ | 0.417^**^ | 0.612^**^ |
| Student-related issues |  |  | 0.274^**^ | 0.411^**^ | 0.566^**^ |
| Workload |  |  |  | 0.448^**^ | 0.535^**^ |
| Social responsibility |  |  |  |  | 0.491^**^ |

∗*p* < 0.05; ∗∗*p* < 0.01; ∗∗∗*p* < 0.001.
