# Supplementary material for: Psychometric Properties of the Effort-Reward Imbalance Questionnaire for Teachers (Teacher ERIQ)
Source: Front Psychol. 2019 Sep 12;10:2047. doi: 10.3389/fpsyg.2019.02047 (PMC6751258; doi:10.3389/fpsyg.2019.02047)
Supplement: Supplementary file 1 [file Data_Sheet_1.zip › tables/TABLE 2 Pearson’s r correlations between the subdimensions of reward.doc]

**TABLE 2** Pearson’s r correlations between the subdimensions of reward

|  | Material reward | Emotional reward | ERI |
| --- | --- | --- | --- |
| Reward | 0.880^**^ | 0.858^**^ | -0.885^**^ |
| Material reward |  | 0.510^**^ | -0.783^**^ |
| Emotional reward |  |  | -0.755^**^ |
