# Supplementary material for: Psychometric Properties of the Effort-Reward Imbalance Questionnaire for Teachers (Teacher ERIQ)
Source: Front Psychol. 2019 Sep 12;10:2047. doi: 10.3389/fpsyg.2019.02047 (PMC6751258; doi:10.3389/fpsyg.2019.02047)
Supplement: Supplementary file 1 [file Data_Sheet_1.zip › tables/TABLE 3 Statistical parameters.doc]

**TABLE 8 Statistical parameters of the ERI scores**

|  | N | Number of items | M | SD | Theoretical score range | Actual score range |
| --- | --- | --- | --- | --- | --- | --- |
| Workload | 442 | 4 | 16.473 | 2.783 | 4-20 | 4-20 |
| Emotional demands | 442 | 3 | 11.502 | 2.453 | 3-15 | 4-15 |
| Student-related issues | 439 | 3 | 11.171 | 2.535 | 3-15 | 3-15 |
| Social responsibility | 439 | 4 | 18.328 | 1.944 | 4-20 | 8-20 |
| Emotional reward | 440 | 7 | 24.211 | 4.172 | 7-35 | 11-35 |
| Material reward | 436 | 7 | 15.748 | 4.624 | 7-35 | 7-33 |
| Effort | 436 | 14 | 57.468 | 7.300 | 14-70 | 37-70 |
| Reward | 436 | 14 | 39.934 | 7.602 | 14-70 | 18-66 |
| ERI | 433 | 28 | 1.519 | 0.474 | 0.2-5 | 0.66-3.89 |
| Overcommitment | 441 | 6 | 24.116 | 3.674 | 6-30 | 12-30 |

M = Mean value, SD = Standard deviation.
