# Supplementary material for: Psychometric Properties of the Effort-Reward Imbalance Questionnaire for Teachers (Teacher ERIQ)
Source: Front Psychol. 2019 Sep 12;10:2047. doi: 10.3389/fpsyg.2019.02047 (PMC6751258; doi:10.3389/fpsyg.2019.02047)
Supplement: Supplementary file 1 [file Data_Sheet_1.zip › tables/TABLE 4 Frequency distribution of ERI scores.doc]

**TABLE 9** Frequency distribution of ERI scores

|  |  | N | % |  |  | N | % |
| --- | --- | --- | --- | --- | --- | --- | --- |
| Social responsibility | high | 411 | 92.6 | Material reward | high | 6 | 1.4 |
|  | low | 1 | 0.2 |  | low | 174 | 39.2 |
| Workload | high | 305 | 68.7 | Emotional reward | high | 92 | 20.7 |
|  | low | 3 | 0.7 |  | low | 15 | 3.4 |
| Emotional demands | high | 250 | 56.3 | Reward | high | 13 | 2.9 |
|  | low | 18 | 4.1 |  | low | 33 | 7.4 |
| Student-related issues | high | 226 | 50.9 | ERI | no | 35 | 7.9 |
|  | low | 20 | 4.5 |  | slight | 212 | 48.0 |
| Effort | high | 274 | 61.7 |  | moderate | 128 | 29.0 |
|  | low | 0 | 0 |  | severe | 58 | 13.1 |
|  |  |  |  | Overcommitment | high | 254 | 57.5 |
|  |  |  |  |  | low | 3 | 0.7 |

high: categories 4 and 5 of response format; low: categories 1 and 2 of response format.

no: ERI ratio<=1; slight: 1< ERI ratio ≤ 1.5; moderate: 1.5 <ERI ratio <=2; severe: ERI ratio>2.
