# Supplementary material for: Psychometric Properties of the Effort-Reward Imbalance Questionnaire for Teachers (Teacher ERIQ)
Source: Front Psychol. 2019 Sep 12;10:2047. doi: 10.3389/fpsyg.2019.02047 (PMC6751258; doi:10.3389/fpsyg.2019.02047)
Supplement: Supplementary file 1 [file Data_Sheet_1.zip › tables/TABLE 5 Associations between ERI and sociodemographic factors.doc]

**TABLE 10** Associations between ERI and sociodemographic factors

|  | **N** | **Workload** | **Social responsibility** | **Emotional demands** | **Student-related issues** | **Effort** | **Material reward** | **Emotional reward** | **Reward** | **ERI** | **Overcommitment** |
| --- | --- | --- | --- | --- | --- | --- | --- | --- | --- | --- | --- |
| **Sex** |  |  |  |  |  |  |  |  |  |  |  |
| male | 122 | 4.238 | 4.663 | 3.943 | 3.837 | 4.221 | 2.13 | 3.399 | 2.762 | 1.616 | 4.065 |
| female | 317 | 4.067 | 4.549 | 3.782 | 3.684 | 4.058 | 2.299 | 3.49 | 2.893 | 1.501 | 4.003 |
| T |  | 2.215^*^ | 2.348^*^ | 1.756 | 1.62 | 2.821^**^ | -2.266^*^ | -1.145 | -2.008^*^ | 2.043^*^ | 0.959 |
| **Age** |  |  |  |  |  |  |  |  |  |  |  |
| <=30(1) | 61 | 3.857 | 4.492 | 3.678 | 3.738 | 3.974 | 3.532 | 2.267 | 2.899 | 1.417 | 3.853 |
| 31-35(2) | 89 | 4.149 | 4.573 | 3.816 | 3.72 | 4.11 | 3.506 | 2.314 | 2.906 | 1.481 | 4.069 |
| 36-40(3) | 178 | 4.16 | 4.596 | 3.867 | 3.7 | 4.12 | 3.424 | 2.229 | 2.828 | 1.552 | 4.08 |
| 41-45(4) | 69 | 4.127 | 4.581 | 3.821 | 3.696 | 4.112 | 3.404 | 2.174 | 2.789 | 1.56 | 3.944 |
| >=46(5) | 45 | 4.233 | 4.67 | 3.97 | 3.853 | 4.207 | 3.489 | 2.302 | 2.88 | 1.535 | 4.022 |
| F |  | 2.713^*^ | 0.934 | 0.958 | 0.306 | 1.413 | 0.696 | 0.551 | 0.676 | 1.201 | 1.992 |
| Post hoc |  | 1＜2,3,4.5 |  |  |  |  |  |  |  |  |  |
| **Types of school** |  |  |  |  |  |  |  |  |  |  |  |
| primary school(1) | 194 | 4.066 | 4.582 | 3.765 | 3.691 | 4.065 | 3.565 | 2.308 | 2.938 | 1.446 | 3.9588 |
| junior high school(2) | 125 | 4.052 | 4.578 | 3.899 | 3.965 | 4.159 | 3.303 | 2.155 | 2.729 | 1.618 | 4.0333 |
| high school(3) | 99 | 4.27 | 4.591 | 3.859 | 3.492 | 4.107 | 3.45 | 2.261 | 2.856 | 1.528 | 4.1347 |
| F |  | 3.381^*^ | 0.022 | 1.109 | 9.327^***^ | 1.227 | 7.598^**^ | 2.047 | 5.726^**^ | 5.084^**^ | 2.699 |
| Post hoc |  | 3＞1,2 |  |  | 2＞1,3 |  | 1＞2 |  | 1＞2 | 2＞1 |  |

Note: the median in the table is the average/number of items in each dimension except ERI ratio.
